# COMMENTARY ON: HOME-BASED TELEREHABILITATION FOR COMMUNITY-DWELLING PERSONS WITH STROKE DURING THE COVID-19 PANDEMIC: A PILOT STUDY

**DOI:** 10.2340/jrm.v56.40662

**Published:** 2024-06-11

**Authors:** Farheen HAIDER, Manju DEVI

**Affiliations:** School of Allied Medical Sciences, Lovely Professional University, Phagwara, Punjab,144411, India

We have read the article by Carl Froilan D. Leochico et al. (1) with great interest but have also found some points that need further clarification.

Enhancing the specificity of the eligibility criteria is crucial, particularly concerning the inclusion criteria. Rather than simply stating “above 18 years”, it is advisable to establish a defined upper age limit for participants. Additionally, it is suggested that the author incorporate information concerning Brunnstrom recovery stages in the inclusion criteria. This would offer clarity regarding the stage at which telerehabilitation would be beneficial. On the other hand, it is important to note that the exclusion criteria should encompass patients with cognitive impairments and those with a history of prior illnesses, as these factors can potentially influence the outcomes. Furthermore, it is advisable for the intervention protocol to adhere to the FIITS principle, which outlines the frequency, intensity, time, and type of exercises. This approach ensures appropriate interventions and facilitates follow-up assessments. Furthermore, it is advisable for the study to incorporate the minimum clinically important difference (MCID) value (2) and to offer detailed information on the reliability and validity of the outcome measurement tools, including the Telepractice Questionnaire, Simple Physical Activity Questionnaire (SIMPAQ), and Happiness Scale.

The introduction of the telepractice questionnaire in the outcome measures and statistical analysis section has raised concerns. As per reference number 11, the questionnaire was initially intended for chronic non-fluent aphasia patients. However, in this study, it was utilized for a non-aphasic population, contradicting the reference. Therefore, it is recommended that this questionnaire be removed. Researchers should prioritize questionnaires with a goal-based design and pay closer attention to their appropriateness for the intended population (3).

The pilot trial in this article deviates from the typical sample size recommendation for pilot studies, which generally suggests 12 to 14 participants, by recruiting 19 participants (4). However, crucial details such as the significance level, data required for determining sample size, assessing data normality, and conducting statistical analysis are missing. Moreover, the study design employed in the pilot experiment is unclear as the sampling strategy is not disclosed. Furthermore, the findings section lacks clarification on data collection methods, particularly for participants who missed sessions. Lastly, the author has not explicitly mentioned the study guidelines followed.

Based on the points discussed above, we recommend that the author exercise caution when determining the sample size, selecting criteria, and utilizing outcome measurement tools, while ensuring proper application of statistical analysis in the manuscript. Despite these considerations, it is noteworthy that this study represents a valuable addition to the limited scientific literature on telerehabilitation in chronic stroke survivors.
