# Supplementary material for: COMMENTARY ON: HOME-BASED TELEREHABILITATION FOR COMMUNITY-DWELLING PERSONS WITH STROKE DURING THE COVID-19 PANDEMIC: A PILOT STUDY
Source: J Rehabil Med. 2024 Jun 11;56:40662. doi: 10.2340/jrm.v56.40662 (PMC11196985; doi:10.2340/jrm.v56.40662)
Supplement: Related texts

# Reply: RESPONSE TO THE “COMMENTARY ON: HOME-BASED TELEREHABILITATION FOR COMMUNITY-DWELLING PERSONS WITH STROKE DURING THE COVID-19 PANDEMIC: A PILOT STUDY”

**Authors:** Carl Froilan D. LEOCHICO

**Affiliations:** 1 Department of Physical Medicine and Rehabilitation, St. Luke’s Medical Center, Global City and Quezon City, Philippines; 2 Department of Rehabilitation Medicine, Philippine General Hospital, University of the Philippines Manila, Manila, Philippines

On behalf of my study team, I am writing in response to the commentary on our article, “Home-based telerehabilitation for community-dwelling persons with stroke during the COVID-19 pandemic: a pilot study", which was published in the Journal of Rehabilitation Medicine in July 2023. I appreciate the opportunity to engage in dialogue and address some of the points raised by Ms Haider and Dr Devi, to whom I would like to express my gratitude for taking the time to read and reflect on our work. Constructive criticism is invaluable in advancing scholarly discourse, and I welcome the chance to clarify and expand upon the ideas presented in our article.

In their commentary, they began with the eligibility criteria. While the upper age limit for study inclusion could have been specified, our team included any adult age (≥18 years) as we did not have prior data on the highest possible age that could participate in a technology-driven intervention. Over the years, Internet penetration has significantly grown in the Philippines (5), which was even tagged as one of the social media capitals of the world. It further increased during the pandemic across different age groups, challenging the stereotype that older adults are hesitant or unwilling to use technology (6, 7). In our study, the participants who passed the eligibility criteria, which implicitly included technology know-how (i.e., using a gadget on their own or assisted by a family member), had a wide age range (i.e. 54.9±11.6 years) (1).

As the study was conducted completely remotely in the context of the COVID-19 pandemic-related restrictions (e.g., physical distancing, suspended in-person outpatient rehabilitation services at our site), we could not perform in-person examinations to accurately obtain additional clinical information, such as muscle tone, that could appropriately inform the participants’ Brunnstrom stages of stroke recovery. Nonetheless, in our eligibility criteria we included only community ambulators, providing us with general information on their functional capacity, which we deemed was relevant to our study methods. In addition, we included only patients without aphasia or significant communication difficulties, inability to follow complex commands, and unstable medical illnesses, which were verified during our pre-intervention video teleconsultation that included mental status examination (1).

In terms of the intervention, we provided details following the FITT principle (frequency, intensity, time, type of exercise) in our article. As indicated in our Methods section and Appendix S1, the participants were instructed to follow our exercise videos for a total of 30 min every other weekday for 2 weeks (i.e., 6 sessions) at their most preferred time (1). For every session, the exercises were composed of warm-up and cool-down periods (approximately 5 min each) and an exercise proper (approximately 20 min), which mainly consisted of simple calisthenics and body conditioning.

We then compared baseline and post-intervention values across certain parameters using remotely administered outcome measures previously reported or validated in the literature. First, we explored the participants’ perception and acceptance of telerehabilitation using our adapted and translated version of the Telepractice Questionnaire, which was developed by Rhodes & Isaki based on prior literature (8). During our protocol development, we were not able to find any standardized tool that could briefly evaluate patients’ perceptions regarding video-based telemedicine familiarity and satisfaction. The frequently used Telehealth Usability Questionnaire (TUQ) and Service User Technology Acceptability Questionnaire (SUTAQ) had more than 20 items each, which we felt might cause survey fatigue among our participants. Hence, we used the existing short and practical Telepractice Questionnaire that was “purpose-built” by the developers for their own study and appropriately aligned with our study aims. Even though the original questionnaire was used for patients with aphasia, it has been successfully used in other non-aphasic and non-stroke populations based on reports published in reputable peer-reviewed journals (9, 10).

Second, we measured the participants’ level of physical activity using the self-reported 5-item Simple Physical Activity Questionnaire (SIMPAQ). The reliability and validity of SIMPAQ have been reported in people with mental illness with substantial representation from limited-resource countries (11). A Stroke Physical Activity Questionnaire (SPAQ) exists, but its psychometric properties (e.g., reliability, concurrent validity) remain to be further evaluated (12).

Lastly, we obtained psychological well-being self-ratings from the participants using a single-item happiness scale (i.e., “On a scale of 0 to 10, with 0 being the saddest and 10 being the happiest, how happy do you feel in general?”). The scale was reported to have good temporal stability and validity, but in a large cohort of students and government employees (13). Nevertheless, there was a study that specifically involved community-dwelling older adults with various diseases, including stroke, that closely looked into such single-item well-being measures, which resulted in significant correlations with happiness, life satisfaction, and quality-of-life scores (14).

While we acknowledge the potential value of reporting the Minimal Clinically Important Difference (MCID) to measure response and provide better interpretability of our findings (rather than merely observing trends from pre- to post-intervention), we could not find established MCID scores for our study outcomes and population. Therefore, this could be an area for research. To the best of our knowledge, MCID estimation has not been given much attention in the context of telerehabilitation, and more so in its application in stroke. Providing MCID estimates may not only differentiate the clinical relevance from the statistical significance of a certain change in an outcome, but it can also assist in determining the effect of a given therapy and designing future clinical trials through effect size estimation and sample size calculation (15).

As with the number of participants in our pilot study, we were able to enrol and analyse the data from 19 participants, which was a little more than the recommended minimum of 12 (3). Pilot studies can have 12 to 35 participants per group for continuous outcomes, although those with a larger sample size can lead to a more precise estimate when planning for the main trial (16). We employed purposive sampling, specifically total enumeration, intentionally recruiting all members (*n*=50) of the stroke support group at our study hospital and providing each with an equal opportunity to be screened for eligibility. As we also aimed to determine the feasibility of our study, we estimated the proportion of people who would be willing to participate, could potentially drop out, and/or could adhere to our recommended intervention. Given our relatively small sample size, we preferred conducting descriptive over inferential statistics as the latter may not yield sound generalizable findings. Hence, we decided to describe trends observed between pre- and post-intervention data.

Lastly, with regard to data collection, three study authors performed outcomes assessment and gathered first-hand information from the participants through phone calls or videoconferencing on social media. Whenever a participant missed a session, we reached out individually through the same methods and collected data accordingly (e.g., reason for missing the session). In the writing of our manuscript, we were guided by the Template for Intervention Description and Replication (TIDieR) checklist to ensure we described our method in adequate detail to allow for its replication (17).

I hope I have been able to enrich some of the concepts in our original article and those raised in the commentary. I am grateful for this opportunity to contribute to this scholarly exchange of ideas. Thank you very much for considering this response.

## References

[1] Leochico CFD, Austria EMV, Gelisanga MAP, Ignacio SD, Mojica JAP. Home-based telerehabilitation for community-dwelling persons with stroke during the COVID-19 pandemic: a pilot study. J Rehabil Med 2023; 55: jrm4405. DOI: 10.2340/jrm.v55.440537427956 PMC10358277

[2] Mouelhi Y, Jouve E, Castelli C, Gentile S. How is the minimal clinically important difference established in health-related quality of life instruments? Review of anchors and methods. Health Qual Life Outcomes 2020; 18: 1–17. DOI: 10.1186/s12955-020-01344-w32398083 PMC7218583

[3] Hajesmaeel-Gohari S, Khordastan F, Fatehi F, Samzadeh H, Bahaadinbeigy K. The most used questionnaires for evaluating satisfaction, usability, acceptance, and quality outcomes of mobile health. BMC Med Inform Decis Mak 2022; 22: 1–9. DOI: 10.1186/s12911-022-01764-235081953 PMC8793175

[4] Julious SA. Sample size of 12 per group rule of thumb for a pilot study. Pharm Stat 2005; 4: 287–291. DOI: 10.1002/pst.185

[5] Ignatius JS, Hechanova MRM. Internet usage from a generational perspective. Philipp J Psychol 2014;47(1):73–92. [accessed 2021 August 31]. Available from: https://www.pap.org.ph/sites/default/files/upload/pjp2014-47-1-pp73-92-ignatiushechanova-internet_usage_from_generational_perspective.pdf

[6] Gallardo MO, Dela Torre J, Ebardo R. The role of initial trust in the behavioral intention to use telemedicine among Filipino older adults. Gerontol Geriatr Med 2024; 10: 23337214231222110. DOI: 10.1177/2333721424125336538196939 PMC10775724

[7] Castillo JMC, Garcia LL, Abalos E, Locsin RC. Living alone and using social media technologies: the experience of Filipino older adults during the COVID-19 pandemic. Nurs Inq 2022; 29: e12460. DOI: 10.1111/nin.1246034541746 PMC8646551

[8] Rhodes NC, Isaki E. Script training using telepractice with two adults with chronic non-fluent aphasia. Int J Telerehabil 2018; 10: 89–104. DOI: 10.5195/ijt.2018.625930588280 PMC6296800

[9] Isaki E, Fangman Farrell C. Provision of speech-language pathology telepractice services using Apple iPads. Telemed J E Health 2015; 21: 538–549. DOI: 10.1089/tmj.2014.015325734686

[10] Leochico CFD, Valera MJS. Follow-up consultations through telerehabilitation for wheelchair recipients with paraplegia in a developing country: a case report. Spinal Cord Ser Cases 2020; 6: 58. DOI: 10.1038/s41394-020-0310-9PMC733850932632104

[11] Rosenbaum S, Morell R, Abdel-Baki A, Ahmadpanah M, Anilkumar TV, Baie L, et al. Assessing physical activity in people with mental illness: 23-country reliability and validity of the simple physical activity questionnaire (SIMPAQ). BMC Psychiatry 2020; 20: 108. DOI: 10.1186/s12888-020-2473-032143714 PMC7060599

[12] Phusuttatam T, Saengsuwan J, Kittipanya-Ngam P. Development and preliminary validation of a Stroke Physical Activity Questionnaire. Stroke Res Treat 2019; 2019: 6764834. DOI: 10.1155/2019/676483431428300 PMC6683788

[13] Abdel-Khalek AM. Measuring happiness with a single-item scale. Soc Behav Pers 2006; 34: 139–150. DOI: 10.2224/sbp.2006.34.2.139

[14] Fukui S, Ishikawa T, Iwahara Y, Fujikawa A, Fujita J, Takahashi K. Measuring well-being in older adults: identifying an appropriate single-item questionnaire. Geriatr Gerontol Int 2021; 21: 1131–1137. DOI: 10.1111/ggi.1429834697875

[15] Rai SK, Yazdany J, Fortin PR, Aviña-Zubieta JA. Approaches for estimating minimal clinically important differences in systemic lupus erythematosus. Arthritis Res Ther 2015; 17: 143. DOI: 10.1186/s13075-015-0658-626036334 PMC4453215

[16] Kunselman AR. A brief overview of pilot studies and their sample size justification. Fertil Steril 2024; 121: 899–901. DOI: 10.1016/j.fertnstert.2024.01.04038331310 PMC11128343

[17] Hoffmann T, Glasziou P, Boutron I, Milne R, Perera R, Moher D, et al. Better reporting of interventions: template for intervention description and replication (TIDieR) checklist and guide. BMJ 2014; 348: g1687. DOI: 10.1136/bmj.g168724609605
